# Deep sequencing reveals microbiota dysbiosis of tongue coat in patients with liver carcinoma

**DOI:** 10.1038/srep33142

**Published:** 2016-09-08

**Authors:** Haifeng Lu, Zhigang Ren, Ang Li, Hua Zhang, Jianwen Jiang, Shaoyan Xu, Qixia Luo, Kai Zhou, Xiaoli Sun, Shusen Zheng, Lanjuan Li

**Affiliations:** 1 State Key Laboratory for Diagnosis and Treatment of Infectious Diseases, Collaborative Innovation Center for Diagnosis and Treatment of Infectious Diseases, The First Affiliated Hospital, School of Medicine, Zhejiang University, Hangzhou 310003, P. R. China; 2 Key Laboratory of Combined Multi-organ Transplantation, Ministry of Public Health; Department of Hepatobiliary and Pancreatic Surgery, The First Affiliated Hospital, School of Medicine, Zhejiang University, Hangzhou 310003, P. R. China; 3 Department of Radiotherapy, The First Affiliated Hospital, School of Medicine, Zhejiang University, Hangzhou 310003, P. R. China

## Abstract

Liver carcinoma (LC) is a common malignancy worldwide, associated with high morbidity and mortality. Characterizing microbiome profiles of tongue coat may provide useful insights and potential diagnostic marker for LC patients. Herein, we are the first time to investigate tongue coat microbiome of LC patients with cirrhosis based on 16S ribosomal RNA (rRNA) gene sequencing. After strict inclusion and exclusion criteria, 35 early LC patients with cirrhosis and 25 matched healthy subjects were enrolled. Microbiome diversity of tongue coat in LC patients was significantly increased shown by Shannon, Simpson and Chao 1 indexes. Microbiome on tongue coat was significantly distinguished LC patients from healthy subjects by principal component analysis. Tongue coat microbial profiles represented 38 operational taxonomic units assigned to 23 different genera, distinguishing LC patients. Linear discriminant analysis (LDA) effect size (LEfSe) reveals significant microbial dysbiosis of tongue coats in LC patients. Strikingly, *Oribacterium* and *Fusobacterium* could distinguish LC patients from healthy subjects. LEfSe outputs show microbial gene functions related to categories of nickel/iron_transport, amino_acid_transport, energy produced system and metabolism between LC patients and healthy subjects. These findings firstly identify microbiota dysbiosis of tongue coat in LC patients, may providing novel and non-invasive potential diagnostic biomarker of LC.

Liver carcinoma (LC) is a common malignancy worldwide, which is associated with high morbidity and mortality^1^. Many studies indicate that a shift in the structure of the intestinal microbial population is involved in the onset and development of chronic inflammatory disease of the liver^2^, liver cirrhosis^3^,^4^ and their complications^5^. The composition of the human intestinal microbiota is closely associated with LC progression through the liver-gut circulation and the intestinal microbiota-liver axis^6^,^7^. We found that in 54% of patients with liver cirrhosis, patient-enriched microbial genes of taxonomically assigned species were of buccal origin, suggesting an invasion of the gut by microbes from the mouth^4^. Therefore, we speculated that the alterations of oral microbiota are associated with chronic inflammatory disease of the liver, including liver cancer. Substantial research suggests that oral microbiome dysbiosis determines health^8^,^9^. For example, a recent study indicates that the salivary microbiota reflects changes in the gut microbiota of patients with cirrhosis with hepatic encephalopathy^10^. However, little is known about the composition of the oral microbiota of patients with LC.

The oral cavity is closely associated with the external environment, and harbours the most diverse microbiome that includes representatives of the phyla Actinobacteria, Bacteroidetes, Firmicutes, Proteobacteria, Spirochaetes, Synergistetes and Tenericutes^8^, some of which occur free in the saliva or form biofilms such as the coat of the tongue and dental plaque. Further, microbiomes differ significantly in different oral habitats such as that between the tongue dorsum and the lateral tongue surface^11^. The microbiome in the biofilm remains relatively stable, particularly in the tongue coat, and is closely associated with oral health and disease^12^. The role of oral microbes in diagnostics^13^ is well established. Thus, clinicians are increasingly using salivary analysis to diagnose systemic disease and monitor general health because of the link between oral and general health^14^.

The tongue is a “mirror” of the body. A principal diagnostic method of Traditional Chinese Medicine (TCM) is the inspection of the tongue, which examines the shape, size, colour, and texture of the tongue body and coat and helps reveal the state of organ function and progression of diseases^15^. Characterization of diverse patterns in the human tongue coat biofilm microbiome may provide useful insights into human health and disease associated with the microbiome. Therefore, more attention should focus on evaluating tongue coat microbial characteristics of patients with LC.

Our aims were to explore the relationship between microbial diversity and LC to contribute to the prevention of LC and to improve patients’ outcomes. For this purpose, here we conducted the first analysis, to our knowledge, of 16S ribosomal RNA gene sequences of microbes present in the tongue coat of patients with LC with cirrhosis.

## Results

### Clinical characteristics of the participants

After strict inclusion and exclusion criteria, this study finally enrolled 35 patients in the early stages of LC patients with cirrhosis who were diagnosed according to the Barcelona Clinic Liver Cancer (BCLC) staging classification as well as 25 healthy subjects. There were no significant differences between the groups in age, sex distribution, and body mass index. We diagnosed 15 of 35 (42.9%) patients and 20 (57.1%) with very early and early stages of LC, respectively. Of these patients, 21 (60%) had <20 ng/ml serum alpha-fetoprotein, and the remainder (40%) had >20 ng/ml. All patients with LC were diagnosed with cirrhosis and were hepatitis B virus soluble antigen (HBsAg)-positive without complications. Serum levels of alanine aminotransferase, aspartate aminotransferase and glutamyltranspeptidase were significantly elevated in patients (all *p* < 0.001). Serum concentrations of albumin and total protein were significantly decreased in the LC patients versus healthy controls (all *p* < 0.001). Serum levels of total bilirubin and direct bilirubin were significantly increased in the patients (*p* = 0.023 and *p* < 0.001, respectively). The number of platelets was significantly reduced in the patients (*p* < 0.001). The details of the clinical characteristics of all subjects are shown in Table 1.

### The increased microbial diversity on the tongue coat in patients with LC

**Figure 1:**
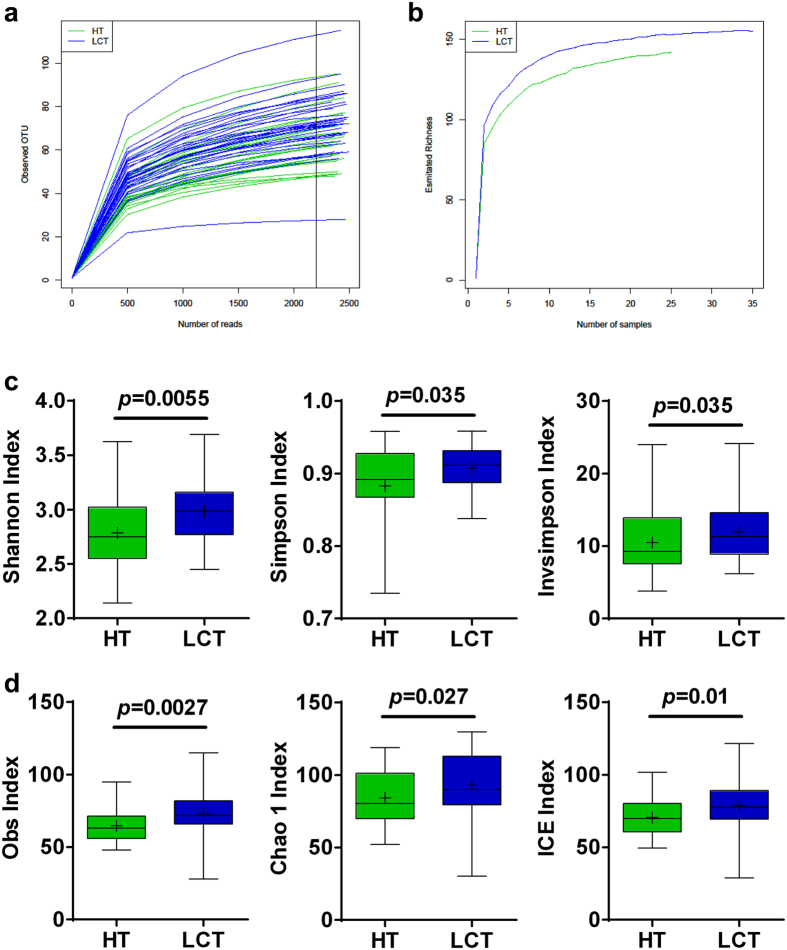
Phylogenetic diversity of tongue coat microbiomes among individuals and between LC patients and healthy subjects. (**a**) Rarefaction analysis of bacterial 16S rRNA gene sequences was used to evaluate if further sequencing would likely detect additional taxa, indicated by a plateau; (**b**) Richness index curves that evaluate the number of samples likely required to identify additional taxa indicated by a plateau; (**c**) Box plots depict microbiomes diversity differences according to the Shannon index, Simpson index and invsimpson index between the LCT and HT; (**d**) Box plots depict microbiomes diversity differences according to the Obs index, Chao 1 index and ICE index between LCT and HT. Box parameters, the “+” symbol represents median value, and the upper and lower ranges of the box represent the 75% and 25% quartiles, respectively. LCT, liver cancer patients tongue coat; HT, healthy subjects tongue coat.

Nine libraries were prepared from a pool of 60 tongue coat microbiomes from the two groups. We used an Illumina MiSeq 2000 System (Shallowater, US) to generate 466,000 filtered sequences. Rarefaction curves of numbers of observed operational taxonomic units (OTUs) per sample showed that the mean number of observed OTUs reached a plateau of approximately 2500 sequence reads (Fig. 1a), and the rarefaction curves of the richness index curves per sample of each of the two cohorts plateaued (Fig. 1b), indicating that approximately all OTUs present in each group were detected and that 2500 reads was sufficient to identify most of the bacterial community members of each tongue coat microbiome. Thus, the OTU table was randomly subsampled at 2500 reads per sample for subsequent community composition analysis. Different diversity indexes (Shannon, Simpson, inverse Simpson [invSimpson], Obs, Chao 1 and Incidence-based Coverage Estimators [ICE]) indicated that the diversity of the LC tongue coating (LCT) microbiome was significantly increased compared with that of the microbiome of healthy subjects (healthy tongue coating, HT) (*p* < 0.05, for all biodiversity parameters) (Fig. 1c,d).

### Principal component analysis (PCA) distinguishes microbiome on tongue coat between LC patients and healthy subjects

**Figure 2:**
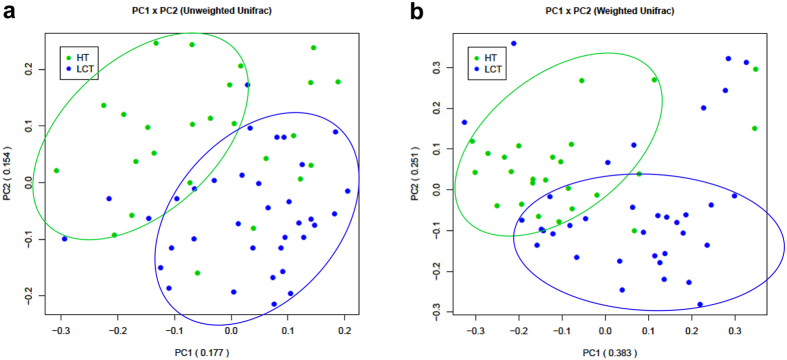
Bacterial diversity clustering by combining unweighted and weighted UniFrac PCoA of tongue coat microbiota. (**a**) Unweighted UniFrac (qualitative); (**b**) Weighted UniFrac (qualitative). Each symbol represents a sample (blue, LCT; green, HT); the variance explained by the PCs is indicated in parentheses on the axes. LCT, liver cancer patients tongue coat; HT, healthy subjects tongue coat.

The unweighted (quantitative) (Fig. 2a) and weighted (qualitative) (Fig. 2b) UniFrac PCA plots, which measure the phylogenetic similarities between microbial communities, shows that the LCT microbiota differs from that of the HT. Similar results were obtained using Principal Coordinate Analysis (PCoA) using the Hellinger distance, Jensen-Shannon Divergence (JSD) analysis, and Spearman coefficient distance methods (Fig. S1).

### Differences in the OTUs of microbiomes between LC patients and healthy subjects

**Figure 3:**
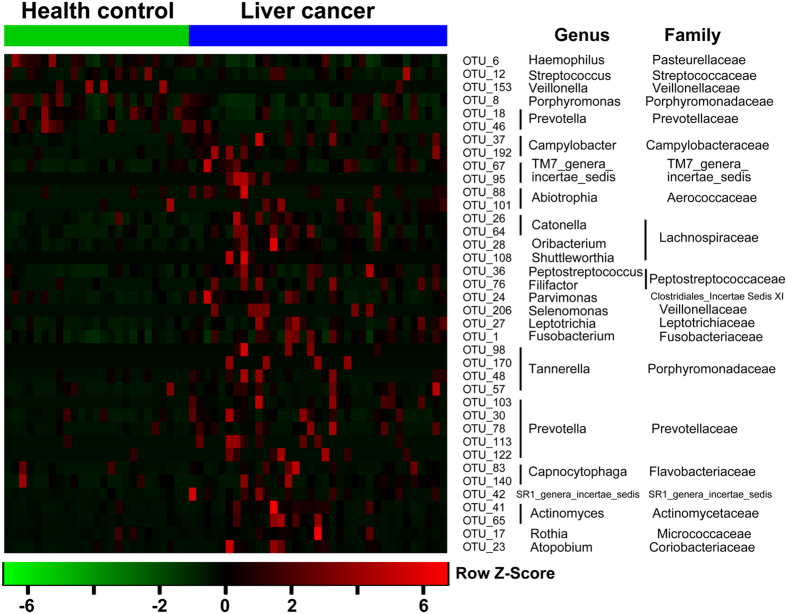
Heat maps of the relative abundances of the discriminatory OTUs that drive the differences between LCT and HT. For each sample, the columns show the relative abundance data of the discriminatory OTUs listed to the right of the figure. The abundances of the genera were clustered using unsupervised hierarchical clustering (blue, low abundance; red, high abundance). The family and genus of each key OTU is noted to the right of the figure. LCT, liver cancer patients tongue coat; HT, healthy subjects tongue coat.

All qualified reads (457,747 [(98.1%]) clustered into qualified OTUs that clustered with randomly chosen qualified reads. Using 97% as the similarity cutoff, 158 qualified species-level OTUs were delineated, while 50 OTUs were discarded because of their low coverage, and 97.6% and 91.6% of all reads were assigned into families and genera, respectively (Supplementary Datasets S1_a,_b). The species-level OTUs and species richness and diversity estimates were obtained for each microbiome (Supplementary Datasets S1_c). The 38 OTUs that were assigned to 23 different genera had significantly different distributions between the LCT and HT microbiomes. Of these OTUs, 6 were decreased, while 32 were increased in the LCT microbiome compared with HT microbiome (Fig. 3).

### Bacterial taxonomic differences between LC patients and healthy subjects

**Figure 4:**
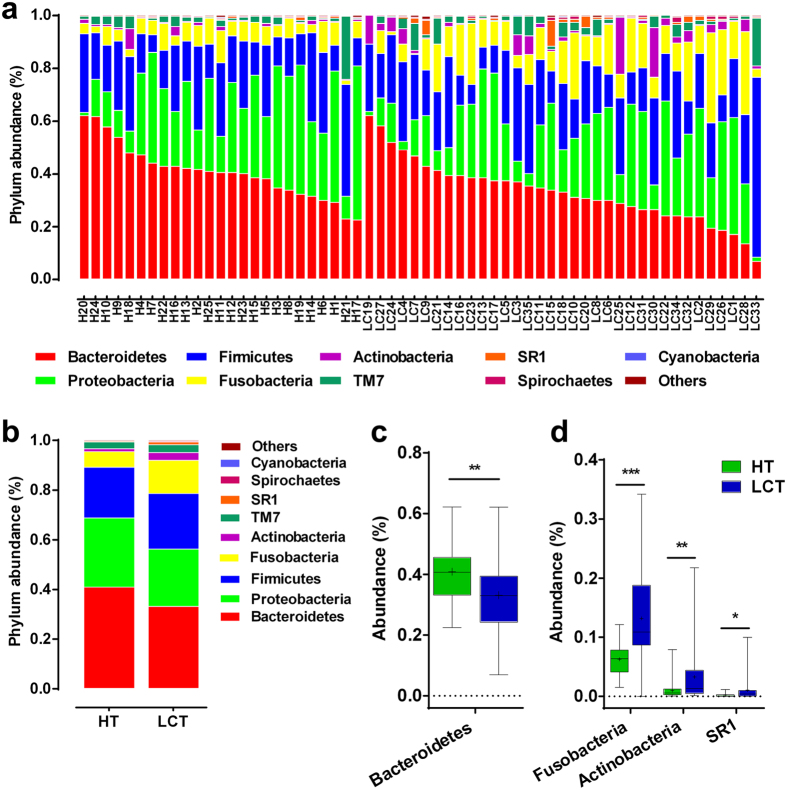
Comparison of phyla of the microbiomes between LCT and HT. (**a**) Comparison of the abundances of bacterial phyla of each sample; (**b**) Comparison of the average abundance of each bacterial phylum in the LCT and HT, respectively; (**c**,**d**) Significant differences among the abundances of discriminatory phyla between LCT (blue) and HT (green); Box parameters, the “+” symbol represents median value, and the upper and lower ranges of the box represent the 75% and 25% quartiles, respectively. p values were calculated using the nonparametric Mann–Whitney test and are shown in Supplementary Datasets S2_a. Significant differences by *p < 0.05; **p < 0.01 and ***p < 0.001. LCT, liver cancer patients tongue coat; HT, healthy subjects tongue coat.

LCT microbial profiles represented 10 phyla (Fig. 4a), and the four predominant (highly abundant and prevalent) phyla were Bacteroidetes, Proteobacteria, Firmicutes and Fusobacteria, which accounted for 90% and 94% of the reads of the LCT and HT samples, respectively (Fig. 4b). Further, 80% of the genera of each microbiome represented *Prevotella*, *Neisseria*, *Fusobacterium*, *Porphyromonas*, *Veillonella*, *Streptococcus* and *Haemophilus* (Fig. S3). Of the 38 OTUs that differed significantly between the LCT and HT microbiomes, 17 and 9 represented Firmicutes and Bacteroidetes, respectively, and the relative abundances of Bacteroidetes were significantly lower in LCT microbiome compared with HT microbiome. Conversely, the differences among the diversities of the Fusobacteria, Actinobacteria and the candidate division SR1 were significantly higher in the LCT microbiome compared with those of the HT microbiome (*p* = 0.006, 0.000, 0.004 and 0.021, respectively) (Fig. 4c,d).

**Figure 5:**
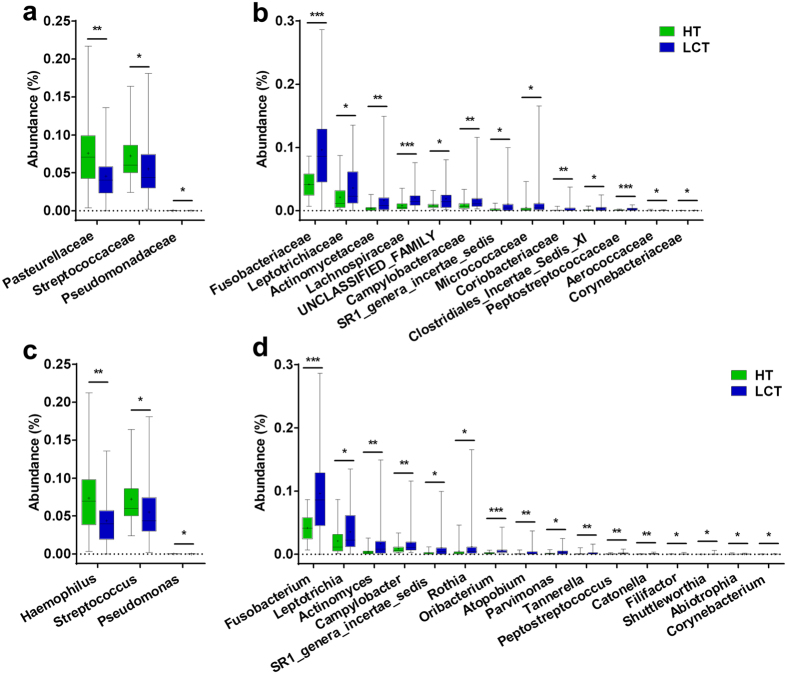
Comparison of the microbiome at the family (**a**,**b**) and genus (**c**,**d**) levels between LCT (blue) and HT (green), respectively. (**a**,**c**) Those enriched in HT; (**b**,**d**) those enriched in LCT; Box parameters, the “+” symbol represents median value, and the upper and lower ranges of the box represent the 75% and 25% quartiles, respectively; *p* values were calculated using the nonparametric Mann–Whitney test and are shown in Supplementary Datasets S2_b (Family) and_c (Genus); significant correlations by *p < 0.05; **p < 0.01 and ***p < 0.001. LCT, liver cancer patients tongue coat; HT, healthy subjects tongue coat.

The differences between LCT and HT microbiomes at the class and order levels are presented in Fig. S2. Consistent with the comparisons of diversities of the two microbiomes, the taxa with increased relative abundance in the LCT group were greater than those with decreased relative abundance. The majority of OTUs that discriminated between the LCT and HT microbiomes were assigned to 16 bacterial families (Fig. 5). Of these discriminatory taxa, *Pasteurellaceae, Streptococcaceae* and *Pseudomonadaceae* were more abundant in the HT microbiome, and 13 were more abundant in the LCT microbiome, including *Fusobacteriaceae, Leptotrichiaceae, Actinomycetaceae* and *Lachnospiraceae* (Fig. 5a,b). Of the 19 discriminatory genera, *Haemophilus*, *Streptococcus* and *Pseudomonas* were strongly enriched in the HT microbiome, and the others were strongly enriched in LCT microbiome, including *Fusobacterium*, *Leptotrichia*, *Actinomyces* and *Campylobacter* (Fig. 5c,d).

### Linear discriminant analysis (LDA) effect size (LEfSe) reveals significant microbial dysbiosis of the tongue coats of patients with LC

**Figure 6:**
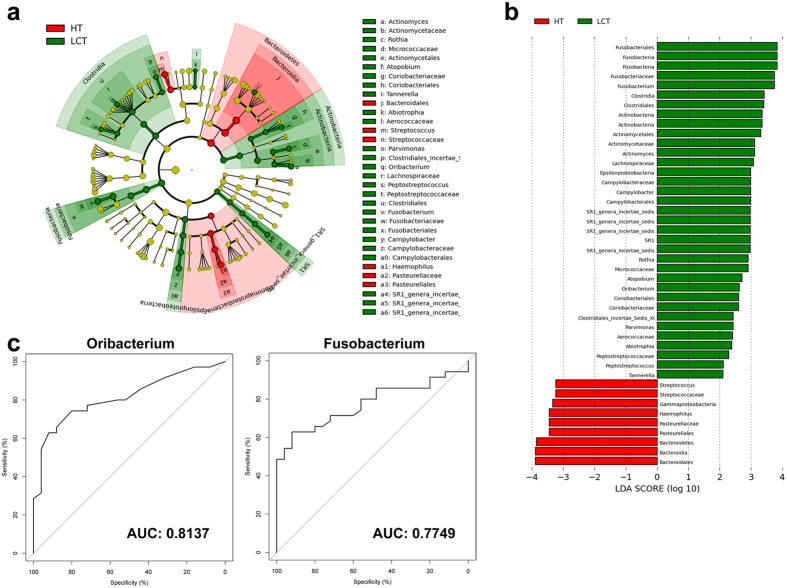
LEfSe and LDA analysis based on OTUs characterize microbiomes between the LCT and HT. (**a**) Cladogram using LEfSe method indicating the phylogenetic distribution of tongue coat microbes associated with patients with LC (green) and healthy subjects (red); (**b**) LDA scores showed the significant bacterial difference between the LCT and HT; (**c**) Prediction of the key genera for LCT from HT; ROC for *Oribacterium*, AUC = 0.8137; ROC for *Fusobacterium*, AUC = 0.8137. LCT, liver cancer patients tongue coat; HT, healthy subjects tongue coat.

We used LEfSe to compare the estimated phylotypes of the LCT and HT microbiota (Fig. 6a,b). The LCT microbiome was characterized by a preponderance of *Fusobacteria*, *Clostridia*, *Actinobacteria*, *Epsilonproteobacteria* and the candidate divisions SR1 (LDA Score (log_10_) > 3), whereas the HT microbiome was characterized by a preponderance of *Bacteroidetes* and *Gammaproteobacteri*a (LDA Score [log_10_] > 3). Two microbial biomarkers (*Oribacterium* and *Fusobacterium*) showed a significant difference in abundance between patients with LC and the HCs (p < 0.001) with ROC (receiver operating characteristic curve)-plot AUC (the area under the parasitemia curve) values of 0.814 and 0.775, respectively (Fig. 6c).

**Figure 7:**
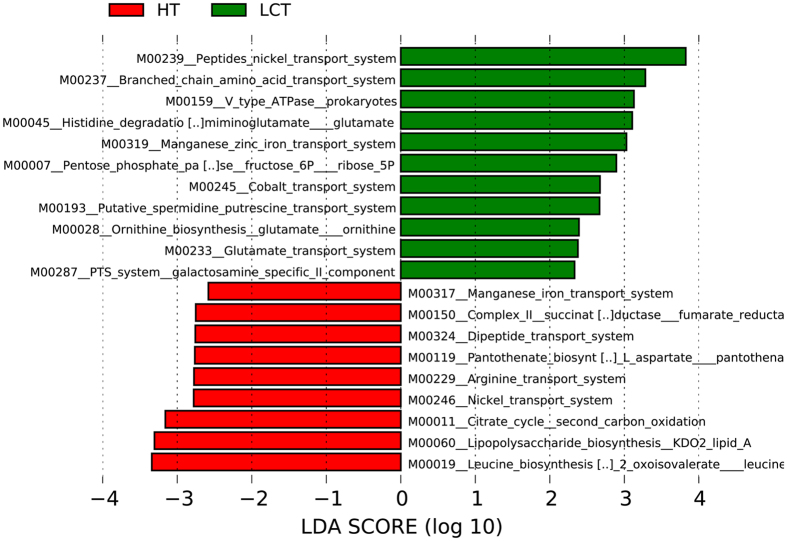
LDA scores predict gene function associated with tongue coat microbiomes in LC patients using PICRUSt. PICRUSt: Phylogenetic Investigation of Communities by Reconstruction of Unobserved States. LCT, liver cancer patients tongue coat; HT, healthy subjects tongue coat.

We next used Phylogenetic Investigation of Communities by Reconstruction of Unobserved States (PICRUSt) (http://picrust.github.com)^16^ software to predict the abundances of gene families in the two microbiomes. The LEfSe outputs show 20 differentially abundant gene families of the LCT and HT microbiomes (Fig. 7), including those microbial gene functions related to the categories nickel/iron_transport, amino_acid_transport and energy produced system and metabolism.

## Discussion

Oral microbiota are attracting increased attention because of their likely associations with cardiovascular disease^17^, rheumatoid arthritis^18^, lung disease^19^, and pancreatic cancers^13^. Moreover, the salivary microbiota reflects changes in the gut microbiota of patients with cirrhosis with hepatic encephalopathy^10^. The correlations of alterations of the gut microbiome with liver cancer are well characterized^6^. Further, one study found that the microbiome of the mouth invades the gut microbiome in patients with liver cirrhosis^4^. In contrast, the characteristics of the tongue coating microbiome of patients with liver cancer are unknown.

Patients with LC tend to have large deposits of tongue coating and a white coat of the posterior two-thirds of the tongue. A white, thick coat represents an imbalance between the rates of epithelial cell proliferation and the removal of dead cells. A decrease in the removal of hydrated dead epithelial cells of the tongue may cause dysbiosis of the tongue coat of patients with LC. Our comparisons here of tongue coat microbial profiles reveal a significant shift in the microbial compositions of the LCT and HT microbiomes. PCA analysis demonstrated that the diversity of the microbiomes of patients with LC were significantly more diverse compared with those of the HT microbiomes. Notably, LCT samples were significantly more diverse compared with HT microbiomes. These data are consistent with findings of increased biodiversity in the salivary microbiota associated with those of patients with other diseases^19^. Using PCoA to cluster sample sets, we detected pronounced differences in the bacterial communities of the LCT and HT microbiomes, suggesting that certain key bacterial species may characterize LCT microbiota.

We found previously in studies of healthy human nasopharynx and salivary microbiota^12^,^20^,^21^ that the dominant phyla in the tongue coat microbiota comprised Bacteroidetes, Proteobacteria, Firmicutes, Fusobacteria and Actinobacteria and that the first four phyla were the most abundant (>90%). The most abundant genera detected in oral microbiota^22^ were present predominantly in the tongue coat microbiome. Further, a significantly higher abundance of the candidate division SR1 in the LCT group detected here suggests that some bacteria present in low abundance may be significantly associated with disease. These results are supplementary to the former healthy oral microbiota data.

Strong evidence suggests that the combined actions of a combination of a series of organism cause disease^8^. Likewise, disease may cause alterations of the compositions of a series of microbial populations. Further, we show here that 38 discriminatory OTUs were detected in the LCT and HT groups, including *Haemophilus*, *Streptococcus*, *Pseudomonas*, *Fusobacterium*, *Leptotrichia*, *Actinomyces*, *Oribacterium* and *Campylobacter*, which were similar to those identified in studies of the salivary microbiomes of cases and controls. Moreover, we detected enrichment of *Leptotrichia*, *Actinomyces*, *Peptostreptococcus* and *Fusobacterium* in the LCT microbiome, which were found previously to dominate the microbial communities of the healthy salivary gland^23^.

Differences in the ecologies of habitats and microbial environments may explain these inconsistent results. For example, *Haemophilus* species correlate negatively with C-reactive protein, a marker of acute inflammation^18^. Several streptococcal species are predominant on the tongue dorsum of healthy subjects^11^, and a meta-analysis found that *Pseudomonas*^24^ is one of the most abundant bacteria supernatants of the saliva of healthy individuals^24^. These three genera were considerably more abundant in the tongue coat microbiomes of healthy controls compared with those of LCT microbiomes. Notably, the enrichment of *Actinomyces* and *Oribacterium* species in patients with rheumatoid arthritis correlates positively with the levels of anticyclic citrullinated peptide^18^, which are present at higher levels in patients with liver carcinoma compared with controls as well as *Peptostreptococcus*^25^ and *Fusobacterium*^22^, which are considerably more abundant in the saliva of patients with disease compared with healthy subjects^12^,^20^,^21^. However, *Fusobacterium* is referred to as a component of the healthy “core microbiome” of the oral cavity^26^. Further, we show here that *Oribacterium* and *Fusobacterium* were highly associated with LC.

Our predictions of the abundance of gene families in tongue coat microbiomes revealed that genes in the categories related to nickel/iron_transport, amino_acid_transport, energy produced system and metabolism differed in abundance between the LCT and HT microbiomes. Shotgun metagenomic analysis should be employed in future studies to reveal the changes in functional genes present in the tongue coat microbiomes of patients with LC and healthy subjects. Such studies may identify the mechanisms of the interaction between the development and progression of liver carcinoma and variations in the tongue coat microbiome.

Human microbiomes are involved in cirrhosis^4^ via body immune mechanism. It is confirmed by mouse models that intestinal bacteria associated with increased risk of LC^27^. Intestinal microbiota disorder promotes the progress of liver cancer, and help cyclophosphamide to shape the anticancer immune response^28^. Moreover, it is reported that gut microbiota may become one of therapeutic targets for LC prevention in advanced liver diseases^29^. These results show that human microbiomes have implications for human liver cancer risk assessment and prevention.

In summary, we present the first description of the microbiome compositions on the tongue coat biofilm in LC patients with cirrhosis. Our results indicate that disease-specific changes in the tongue coat biofilm microbiome occur in patients with LC. Among 38 LC-discriminatory OTUs, the *Oribacterium* and *Fusobacterium* distinguished between LC patients and health controls. These findings therefore identify dysbiosis of the LC tongue coat microbiota and provide novel and non-invasive diagnostic biomarkers of liver carcinoma.

## Material and Methods

### Study design and enrolled patients

All enrolled individuals had healthy oral tissues and gingiva. A dentist determined the oral health of all individuals. The dentist performed a full clinical examination of the mouth that included inspection of the teeth, oral mucosa and periodontal tissues. All participants had normal oral mucous membranes and were free from non-restored carious lesions. At most sites, periodontal tissues showed no clinical signs of inflammation such as redness, swelling, or bleeding on probing and were judged free of gingivitis or periodontitis.

Liver cancer patients with cirrhosis were initially diagnosed according to international guidelines by analysis of imaging studies, clinical symptoms, physical signs, laboratory tests and medical histories. Diagnosis was confirmed by histopathological examination following surgery or percutaneous ultrasound-guided biopsy. LC was staged according to the BCLC criteria^30^,^31^. The degree of liver cancer differentiation was determined using the Edmondson grading system^32^. Liver function was assessed according to the Child-Pugh scoring system. We excluded those who were diagnosed with intrahepatic cholangiocarcinoma or underwent prior anticancer treatment. The patients were co-infected with HCV, HIV or other secondary organism (bacteria and fungi) infection were also excluded. Finally, we screened and included 35 patients with early LC patients with cirrhosis.

We enrolled 25 matched healthy volunteers who met the inclusion criteria described in our previous study^4^. Healthy volunteers were in the normal ranges of physical examinations; liver biochemistry tests; routine blood, urine and stool examinations; serological tests (including detection of HBsAG, and antibodies against hepatitis C virus, *Treponema pallidum*, and human immunodeficiency virus); liver and renal function tests; electrolyte concentrations; liver ultrasound findings and electrocardiogram and chest X-ray results. Exclusion criteria for volunteers included hypertension, diabetes, obesity, metabolic syndrome, irritable bowel syndrome, non-alcoholic fatty liver disease, coeliac disease and liver cirrhosis. All enrolled individuals who received antibiotics, probiotics or both within 8 weeks before enrolment, or consumption of unhealthy substances (including alcohol, cigarette/tobacco and drug abuse) were also excluded.

Written informed consent and questionnaires addressing previous and current diseases, lifestyles and medication (Table S1) were obtained from all subjects who voluntarily provided samples. The study was approved by the Ethics Committee of the First Affiliated Hospital, School of Medicine, Zhejiang University (reference number: 2014-335). The methods were carried out in accordance with the national guidelines, as we previously performed^3^,^4^.

### Sample collection and DNA extraction

Before sampling, the subjects were asked to rinse and gargle twice with sterile water and extend their tongue as far as possible. The tongue was divided into six functional segments according to Winkel^33^, and a professional stomatologist used a tongue scraper to collect the tongue coat from the posterior middle area to the anterior middle area^34^. The coat samples were immersed in phosphate-buffered saline, transferred to the laboratory, shaken, centrifuged immediately, and the supernatant was discarded. The pellets were stored at −80 °C within 1 hour.

Microbial DNA on the tongue coat was extracted using a Qiagen Mini Kit (Qiagen, Hilden, Germany) following the manufacturer’s instructions as described previously^3^,^35^,^36^. The DNAs were quantified using a Qubit 2.0 Fluorometer (Invitrogen, Carlsbad, CA, USA), and molecular size was estimated using agarose gel electrophoresis. All tongue coat microbial DNAs were diluted to 10 ng/μL for microbial analysis.

### PCR and sequencing

The universal target V3–V4 region of the 16s rRNA gene was PCR-amplified using primers 338F 5′-barcode-ACTCCTACGGGAGGCAGCA-3′ and 806R 5′-GGACTACHVGGGTWTCTAAT-3′, where barcode is an eight-base sequence unique to each sample. PCR reactions contained 4 μL of 5× Fast Pfu Buffer (TransGen Biotech, Beijing, China), 2 μL of 2.5 mM dNTPs, 0.8 μL of each primer (5 μM), 0.4 μL of Fast Pfu Polymerase, and 10 ng of template DNA. Four PCR reactions were run for each sample in a thermocycler (Eppendorf Mastercycler) as follows: 95 °C for 2 min, followed by 25 cycles at 95 °C for 30 s, 55 °C for 30 s, 72 °C for 45 s and a final extension at 72 °C for 10 min. PCR reactions from the same sample were pooled, purified using agarose gel separation and extraction (Axygen Biosciences, Union City, CA, USA) and quantified using a fluorometric kit (Quant-iT PicoGreen, Invitrogen). Purified amplicons were pooled in equimolar amounts for library preparation. The construction of sequencing libraries and paired-end sequencing (2 × 250 bp) was performed using the MiSeq System (Illumina) at the State Key Laboratory for Diagnosis and Treatment of Infectious Diseases (Zhejiang University, Hangzhou, China) according to standard protocols. The raw reads were deposited into the European Nucleotide Archive database (Study accession Number: PRJEB12503, Second study accession number: ERP013989).

### Sequence assembly and analysis

PCR primers incorporated sample-specific barcodes for multiplex sequencing using the Illumina MiSeq System (paired-end 250-nt reads). Paired V4-16S rRNA sequences were trimmed to 200 bp and merged into a single sequence using FLASH v1.2.10 software. Merged sequences were filtered to exclude low-quality reads and binned according to their specific barcodes. Sequences were assembled according to the criteria as follows: (1) Ambiguous bases (N) and mismatches in barcode and primer regions were not allowed; (2) the maximum mismatch rate in the overlap region was 0.05; (3) maximum length and minimum length of reads (without barcodes and primers) were 100 nt and 500 nt, respectively. Chimeras were removed using USEARCH software. The sample size of each sample was equalized by random subtraction to 2500 reads. The remaining effective sequences were binned into OTUs using USEARCH software with a cutoff of 97% identity^37^. For each OTU, reads with the highest frequencies were chosen as representative sequences.

Representative sequences were assigned at different taxonomic levels (from phylum to genus) to the bacterial SILVA dataset following the Bayesian approach with a 97% cutoff value^38^. Bacterial diversity was determined using sampling-based analysis of OTUs and is displayed as a rarefaction curve. Bacterial richness and diversity across the samples were calculated using the indexes as follows: Chao 1, Obs, ICE, Simpson, invSimpson and Shannon, which were estimated at a distance of 3%. Both variants of Simpson’s index are based on D = sum p_i^2^. Simpson returns values of 1–D and invSimpson returns values of 1/D. PCA using weighted and unweighted UniFrac distance metrics was conducted using a random sample of 2,500 sequences per samples, and the R package (http://www.R-project.org/) was used to visualise the interactions among the bacterial communities of different samples.

To reduce the possibility of PCoA calculation errors, we used three different calculation methods, including the Hellinger distance, JSD analysis and the Spearman coefficient distance to conduct distances analysis using a custom R program function provided by the EBML (http://enterotype.embl.de/enterotypes.html#dm). The heat map of key variables was generated using Heatmap Builder. Sequences were annotated using the Ribosomal Database Project version 2.4 classifier with a confidence level = 0.5^39^. LEfSe (http://huttenhower.sph.harvard.edu/galaxy/) was used to identify taxa that differed consistently between sample types according to a published study^40^. LEfSe was used to identify biomarkers of microbiomes in tongue coat samples of the LCT group and those of healthy controls (HT) at multiple levels in datasets, grade the biomarker according to statistical significance, and visualise the results using taxonomic bar charts and cladograms^41^. Gene family abundance in the tongue coat microbiome was predicted using PICRUSt.

### Statistical analysis

GraphPad Prism V.6.0 (San Diego, CA, USA) was used for all analyses and preparation of graphs. The results are expressed as the mean ± SEM, and statistical analyses were performed using the two-tailed non-parametric Kruskal–Wallis test to evaluate the significance of differences in microbial taxa, clinical measures and diversity indexes. Differences with a *p* value < 0.05 were considered significant. The Wilcoxon rank sum test was used to compare the crucial taxa between groups. To identify biomarkers in the LCT microbiome, a receiver operating characteristic (ROC) curve for each crucial taxa was generated, and the area under the parametric curve (AUC) was computed by numerical integration using the R software pROC package (10,000 bootstrap replicates). Validated tongue coat biomarkers were fit to logistic regression models, and the sensitivity and specificity of biomarker combinations were estimated by identifying the cutoff point of the predicted probability that yielded the highest sum of sensitivity and specificity according to published studies^13^.

## Additional Information

**How to cite this article**: Lu, H. *et al.* Deep sequencing reveals microbiota dysbiosis of tongue coat in patients with liver carcinoma. *Sci. Rep.* **6**, 33142; doi: 10.1038/srep33142 (2016).

## Supplementary Material

Supplementary Information

Supplementary Dataset S1

Supplementary Dataset S2

## Figures and Tables

**Table 1:** Clinical characteristics for all participants.

		Liver cancer patients		Healthy controls		P
Clinical and pathological Indexes		N = 35	%	N = 25	%	
Age (year)		50.30 ± 5.86		48.20 ± 6.03		0.19*
Gender	Female	5	14.3	5	20.0	0.728#
	Male	30	85.7	20	80.0	
BMI (kg/m^2^)		22.52 ± 1.55		22.56 ± 1.63		0.926
BCLC	0 (very early)	15	42.9			
	A (early)	20	57.1			
AFP (ng/ml)	≤20	21	60.0	25	100	<0.001
	>20	14	40.0	0	0	
Tumor size (cm)	≦2	15	42.9			
	2< & ≦5	20	57.1			
Tumor number	Single	34	97.1			
	Multiple	1	2.9			
Tumor differentiation	I-II	26	74.3			
	III-IV	9	25.7			
Liver cirrhosis	Yes	35	100			
CTP score	A	35	100			
HBsAg	Positive	35	100			
Mild complication	No	35	100			
ALT (U/L)		37.24 ± 22.61		19.36 ± 6.85		<0.001
AST (U/L)		34.51 ± 12.88		20.60 ± 4.95		<0.001
GGT (U/L)		53.00 ± 26.48		18.00 ± 9.29		<0.001
Total protein (g/L)		61.99 ± 5.72		75.12 ± 3.79		<0.001
Albumin (g/L)		34.18 ± 2.87		49.03 ± 2.17		<0.001
Globulin (g/L)		26.59 ± 3.23		26.10 ± 2.96		0.697
Total bilirubin (μmol/L)		17.69 ± 7.88		13.48 ± 4.89		0.023
Direct bilirubin (μmol/L)		7.51 ± 4.27		4.60 ± 1.73		<0.001
Indirect bilirubin (μmol/L)		10.10 ± 4.17		8.88 ± 3.48		0.240
Platelet (10E9/L)		128.97 ± 65.65		225.84 ± 63.98		<0.001
CA 199 (U/ml)		11.01 ± 7.62		8.09 ± 6.33		0.132
CA 125 (U/ml)		12.32 ± 6.75		9.10 ± 4.27		0.054

The continuous variables were presented as mean ± SD.

Abbreviations: BMI, body mass index; AFP, alphafetoprotein; HBsAg, hepatitis B surface antigen; ALT, alanine aminotransferase; AST, aspartate aminotransferase; GGT, glutamyl transpeptidase; CTP score, Child-Turcotte-Pugh score; CA 199, carbohydrate antigen 19–9; CA 125, carbohydrate antigen 12–5.

^*^Independent t-test.

^#^Pearson Chi-Square test or Fisher exact test.
