# Supplementary material for: Deep sequencing reveals microbiota dysbiosis of tongue coat in patients with liver carcinoma
Source: Sci Rep. 2016 Sep 8;6:33142. doi: 10.1038/srep33142 (PMC5015078; doi:10.1038/srep33142)
Supplement: Supplementary Information [file srep33142-s1.doc]

**Deep sequencing reveals microbiota dysbiosis of tongue coat in patients with liver carcinoma**

Haifeng Lu ^1ǂ^, Zhigang Ren ^1,2ǂ^, Ang Li ^1^, Hua Zhang ^1^, Jianwen Jiang ^1,2^, Shaoyan Xu ^2^, Qixia Luo ^1^, Kai Zhou ^1^, Xiaoli Sun^3^, Shusen Zheng^1,2^ & Lanjuan Li^1*^

^ǂ^Haifeng Lu and Zhigang Ren contributed equally to this work.

^1^State Key Laboratory for Diagnosis and Treatment of Infectious Diseases, Collaborative Innovation Center for Diagnosis and Treatment of Infectious Diseases, The First Affiliated Hospital, School of Medicine, Zhejiang University, Hangzhou 310003, P.R. China.

^2^Key Laboratory of Combined Multi-organ Transplantation, Ministry of Public Health; Department of Hepatobiliary and Pancreatic Surgery, The First Affiliated Hospital, School of Medicine, Zhejiang University, Hangzhou 310003, P.R. China.

^3^Department of Radiotherapy, The First Affiliated Hospital, School of Medicine, Zhejiang University, Hangzhou 310003, P.R. China.

**Supplementary information**

**Supplementary table 1**. Questionnaire: Data for subjects participating in the investigation of the tongue coat microbiome

**Translated from Chinese**

**Data for subjects participating in the investigation of the tongue coat microbiome**

**Name： sex：**F/M **Date of birth (**month-year**)：**

**Telephone number(s)： Sample NO:**

| Data of follow-up(Y-M-D) | | |  | | |
| --- | --- | --- | --- | --- | --- |
| Way of follow- up | | | 1. outpatient 2 inpatient | | |
| A survey   of disease symptom | 1.Liver, lung or previous related surgery  2.Basic diseases such as diabetes, coronary disease,hypertension  3. Ascites  4.Gastrointestinal bleeding  5.Liver cirrhosis  6.Co-infection with HCV, HIV or other secondary bacterial infection  7.Symptoms of respiratory: shortness of breath, sputum production,wheezing or chest pain or discomfort.  8.Oothers | |  | | |
| X-ray examation | | | Examation day： | | |
|  |  |  |  |  |  |
| Oral Health Screening | | | Examation day： | | |
|  |  |  | 1. [periodontitis](http://dict.youdao.com/w/periodontitis/) □  2. oral ulcer □  3. Cavities □  4. Tongue coat:  5. others: ______ | | |
| Biochemical blood routine inspection | | | Examation day： | | |
|  |  |  |  |  |  |
| Physical sign | Blood pressure（mmHg） | |  | | |
|  | Weight（kg） | |  | | |
|  | [Height](javascript:void(0);) (cm) | |  | | |
|  | Body mass index （BMI） | |  | | |
|  | Others | |  | | |
| Lifestyles | Daily consumption of cigarettes | |  | | |
|  | Daily consumption of  alcohol | |  | | |
|  | Others (including food habits and other drugs) | |  | | |
|  | Fitness habits | | Form: Time: | | |
|  | Dietaryhabits | Yoghurt |  | | |
|  |  | staple |  | | |
|  | Psychological quality | | 1.better 2.good 3.worse □ | | |
|  | Treatment compliance | | 1.better 2.good 3.worse □ | | |
| Drugs | Antibiotics | |  | | |
|  | Probiotics | |  | | |
|  | Or others | |  | | |
|  | Drugs 1: (name) | |  | | |
|  | Drug dosage | | Times/Day: Dosage/time: mg | | |
|  | Drugs 2:(name) | |  |  |  |
|  | Drug dosage | | Times/Day: Dosage/time: mg | | |
|  | Drugs 3:(name) | |  | | |
|  | Drug dosage | | Times/Day: Dosage/time: mg | | |
|  | Others | |  | | |
| Other need to supplement | | |  | | |
| Patients signature | | |  | | |
| Doctor signature | | |  | | |

**Form in Chinese**

**肝硬化患者口咽部微生态随访服务记录表**

**姓名： 性别： 年龄：　　联系方式： 标本编号**

| 随访日期 | | | 年 月 日 | |  | |  | |  | |
| --- | --- | --- | --- | --- | --- | --- | --- | --- | --- | --- |
| 随访方式 | | | 1门诊 2住院□ | | 1门诊 2住院 | | 1门诊 2住院 | | 1门诊 2住院 | |
| 病情调查 | 1手术  2糖尿病、冠心病、高血压等基础性疾病  3 腹水  4 胃肠道流血  5肝硬化  6感染  7 呼吸道感染症状：是否有痰，呼吸是否短促，胸部伴疼痛或不适等  8 其它 | |  | |  | |  | |  | |
| 生化  血常规 | | | 检查日期： 月 日 | | 检查日期： 月 日 | | 检查日期： 月 日 | | 检查日期： 月 日 | |
|  |  |  |  |  |  |  |  |  |  |  |
| 胸片等检查 | | | 检查日期： 月 日 | | 检查日期： 月 日 | | 检查日期： 月 日 | | 检查日期： 月 日 | |
| 口腔疾病筛查 | | | 检查日期： 月 日 | | 检查日期： 月 日 | | 检查日期： 月 日 | | 检查日期： 月 日 | |
|  |  |  | 1. 牙周炎 2. 口腔溃疡 3. 蛀牙 4. 其它:_____   □ | | 1. 牙周炎 2. 口腔溃疡 3. 蛀牙 4. 其它:_____   □ | | 1. 牙周炎 2. 口腔溃疡 3. 蛀牙 4. 其它:_____   □ | | 1. 牙周炎 2. 口腔溃疡 3. 蛀牙 4. 其它:_____   □ | |
| 体  征 | 血压（mmHg） | |  | |  | |  | |  | |
|  | 体重（kg） | |  | | / | | / | | / | |
|  | 身高(cm) | |  | |  | |  | |  | |
|  | 体质指数 | |  | |  | |  | |  | |
|  | 其 他 | |  | |  | |  | |  | |
| 生  活  方  式  调查 | 日吸烟量 | | 支 | | 支 | | /　　 支 | | /　　 支 | |
|  | 日饮酒量 | | 两 | | 两 | | 两 | | 两 | |
|  | 健身运动 | | 类型：  次/周 分钟/次 | | 类型：  次/周 分钟/次 | | 类型：  次/周 分钟/次 | | 类型：  次/周 分钟/次 | |
|  | 饮食习惯 | 酸奶 |  | |  | |  | |  | |
|  |  | 主食 |  | |  | |  | |  | |
|  | 心理调整 | | 1良好 2一般 3差 □ | | 1良好2一般 3差 □ | | 1良好2一般 3差 □ | | 1良好 2一般 3差 □ | |
|  | 遵医行为 | | 1良好 2一般 3差 □ | | 1良好2一般 3差 □ | | 1良好2一般 3差 □ | | 1良好 2一般 3差 □ | |
| 用  药  情  况  调  查 | 抗生素药物 | |  | |  | |  | |  | |
|  | 益生菌制剂 | |  | |  | |  | |  | |
|  | 或其它药物 | |  | |  | |  | |  | |
|  | 服药依从性 | | 1规律2间断3不服药□ | | 1规律2间断3不服药□ | | 1规律2间断3不服药□ | | 1规律2间断3不服药□ | |
|  | 药物名称1 | |  | |  | |  | |  | |
|  | 用 法 | | 每日 次 | 每次 mg | 每日 次 | 每次 mg | 每日 次 | 每次 mg | 每日 次 | 每次 mg |
|  | 药物名称2 | |  | |  | |  | |  | |
|  | 用 法 | | 每日 次 | 每次 mg | 每日 次 | 每次 mg | 每日 次 | 每次 mg | 每日 次 | 每次 mg |
|  | 药物名称3 | |  | |  | |  | |  | |
|  | 用 法 | | 每日 次 | 每次 mg | 每日 次 | 每次 mg | 每日 次 | 每次 mg | 每日 次 | 每次 mg |
|  | 其他 | |  | |  | |  | |  | |
| 其它需要补充说明的情况 | | |  | |  | |  | |  | |
| 患者签名 | | |  | |  | |  | |  | |
| 医生签名 | | |  | |  | |  | |  | |
| 下次随访日期 | | | x年x月x日 | |  | |  | |  | |

**Supplementary Figures S1-S3**.

**Figure S1.** Principal Coordinate Analysis (PCoA) using unweighted UniFrac scores of microbiomes of LCT (blue) and HT (green) samples using different methods for calculating distances as follows: Hellinger distance, Jensen-Shannon Divergence (JSD) analysis and the Spearman coefficient distance. Each symbol represents a sample (green, LCT; red, HT); the variance explained by the PCoA is indicated in parentheses on the axes.

**
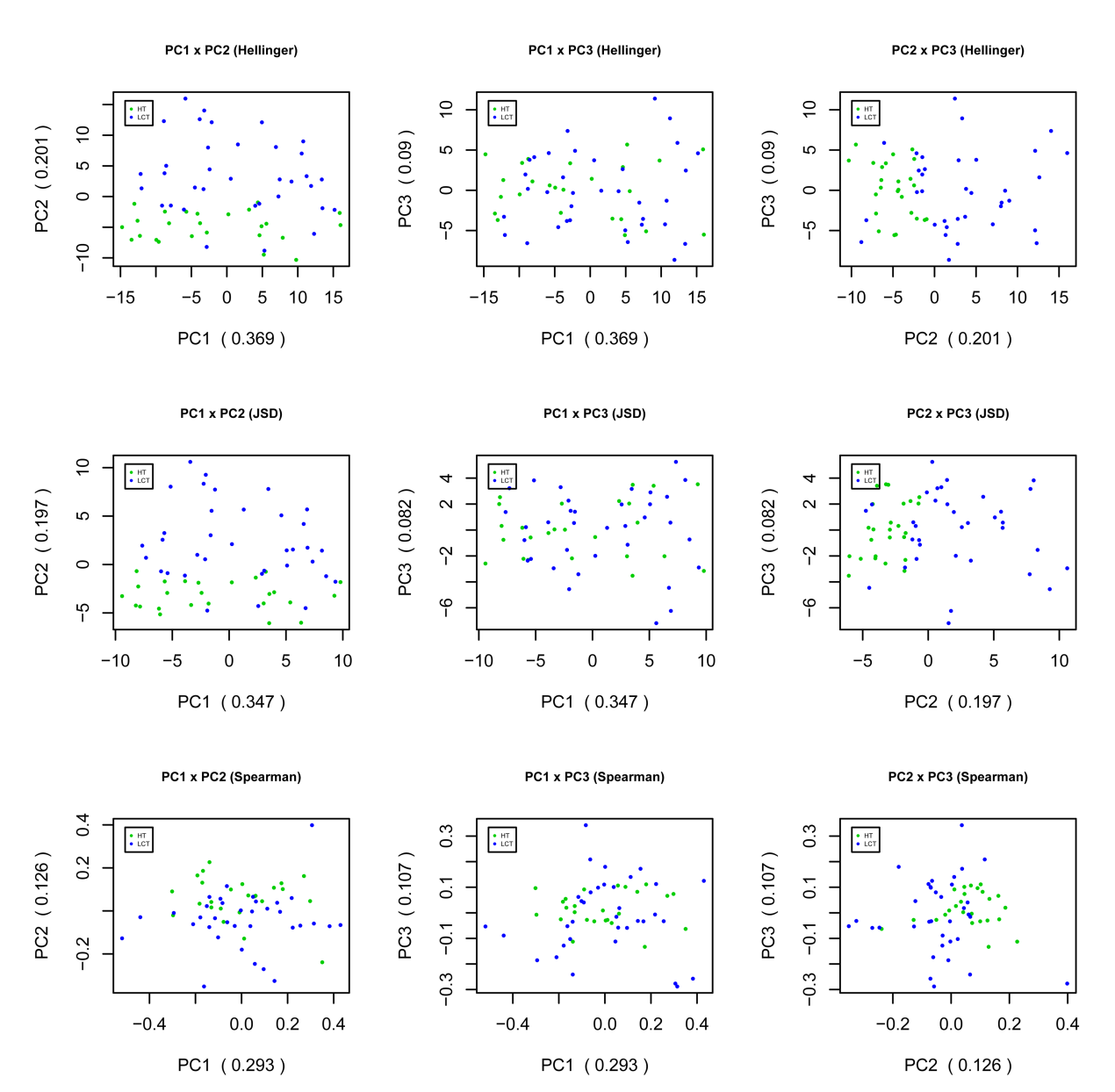
**

**Figure S2.** Comparison of microbiomes according to class (a, b) and order (c, d) between LCT (blue) and HT (green) microbiomes, respectively; a and c, enriched in HT; b and d, enriched in LCT; Box parameters, the bold line represents median diversity, and the upper and lower ranges of the box represent the 75% and 25% quartiles, respectively; The *p* values were calculated using the nonparametric Mann–Whitney test shown in Supplementary Datasets S2_d (Class) and _e (Order); significant correlations indicated as **p* < 0.05; ^**^*p* < 0.01 and ^***^*p* < 0.001.


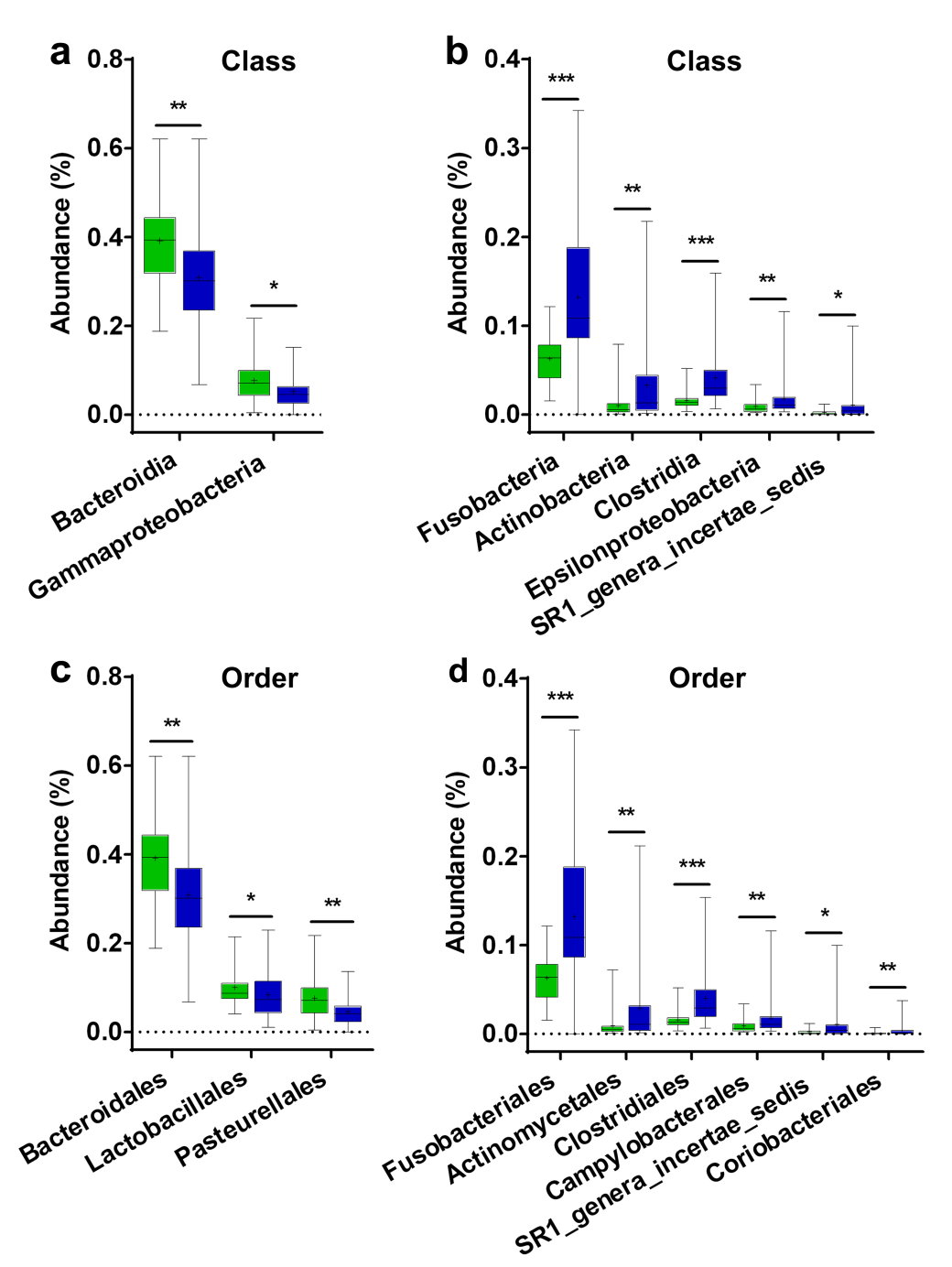


**Figure S3.** Bar plot of the most abundant bacterial genera in each sample.


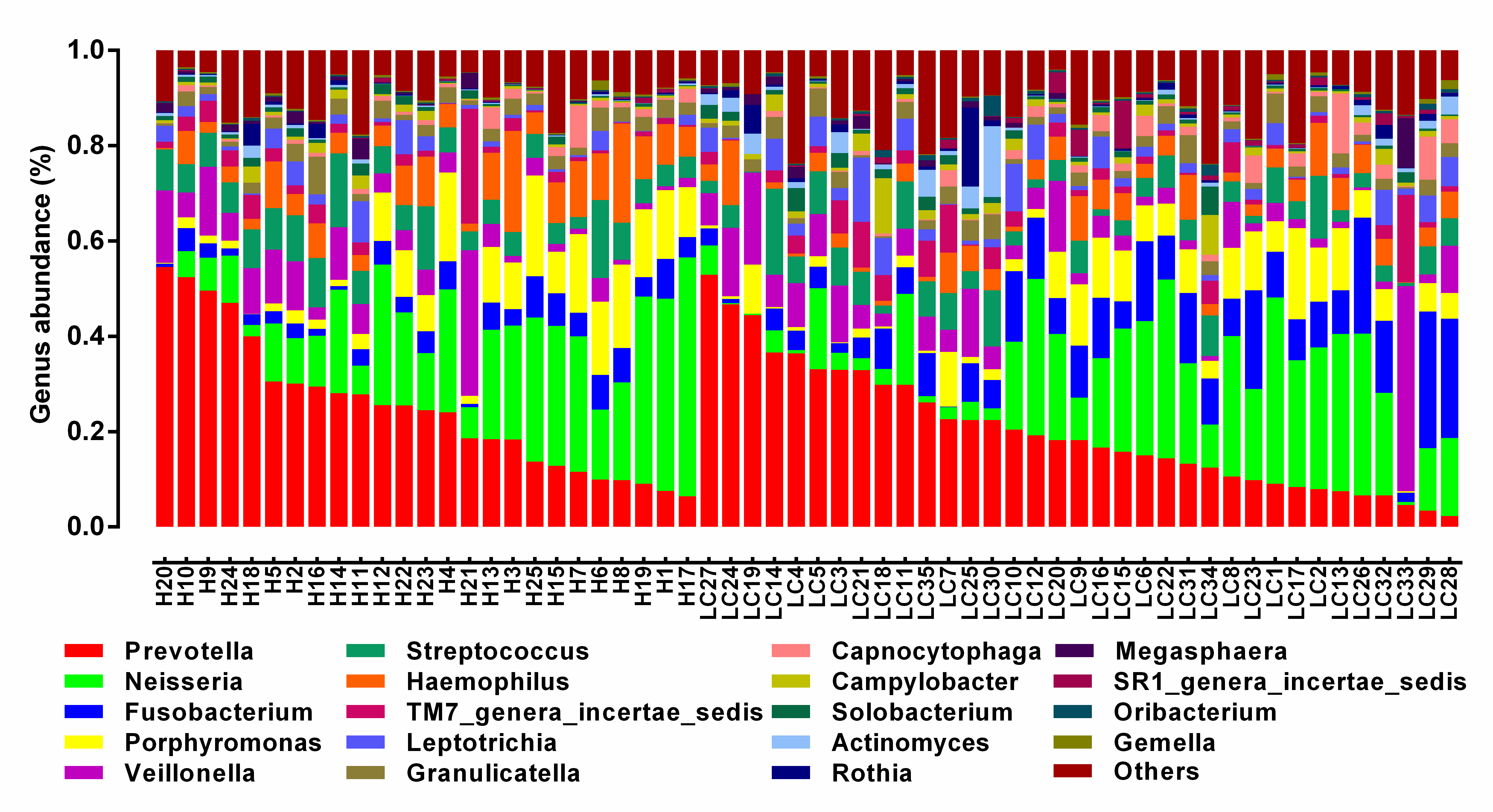


**Supplementary Datasets S1.** Sequences determined using the MiSeq System (Illumina). Supplementary Datasets S1_a, Relative abundance of OTUs present in >5 samples; Supplementary Datasets S1_b, Annotation of all OTU detected using 16-s rDNA gene sequencing; Supplementary Datasets S1_c, Species-level OTUs and species richness and diversity estimates of each microbiome.

**Supplementary Datasets S2.** Statistical analysis of tongue coat microbiomes using the nonparametric Mann–Whitney test for phyla (Supplementary Datasets S2_a), classes (Supplementary Datasets S2_b), orders (Supplementary Datasets S2_c), families (Supplementary Datasets S2_d) and genera (Supplementary Datasets S2_e).
